# Uptake of signposting to web-based resources: pregnant women’s use of a preventive web-based intervention

**DOI:** 10.1186/s12875-023-02130-5

**Published:** 2023-09-16

**Authors:** Emil Rønn Sørensen, Ida Scheel Rasmussen, Gritt Overbeck, Volkert Siersma, Clara Lundmark Appel, Philip Wilson

**Affiliations:** 1 The Research Unit for General Practice and Section of General Practice, Department of Public Health, University of Copenhagen, Copenhagen, Denmark; 2 Centre for Rural Health, Institute of Applied Health Sciences, University of Aberdeen, Aberdeen, Scotland

**Keywords:** Pregnancy, Internet-based intervention, Health promotion, Prenatal care, Mental health, Primary health care, E-health

## Abstract

**Background:**

Signposting to web-based interventions is becoming increasingly popular in primary care. Most resources are focused on individuals with clinical problems, but less is known about the uptake of general practice (GP) signposted web-based interventions. GPs in Denmark are responsible for scheduled preventive care during pregnancy and the child’s first five years. In the “Family Well-being in General Practice” trial the web-based intervention “Resilientchild.dk” is introduced at these consultations. Resilientchild.dk is designed to improve the capacity of parents to understand the mental state of themselves, their partners, and their children. In this study we assess the uptake and use of this web-based intervention.

**Objective:**

To describe participant and practice characteristics associated with the use of a web-based psychoeducational intervention. Eligible participants were pregnant women presenting at their first antenatal assessment, usually around 6–10 gestational weeks.

**Methods:**

The study was nested in a cluster randomised trial of resilientchild.dk. We conducted a relative importance analysis, which allows for determination of the variables most strongly associated with website use. To assess the direction and magnitude of the influences of the identified variables, we applied multinomial generalized linear mixed modelling. A practice random effect allows us to account for clustering of women within practices.

**Results:**

Parity and the absence of a nurse or midwife in the practice were important factors driving a decrease in the likelihood of using resilientchild.dk. Being a student or living outside the capital city were important factors driving an increase in the likelihood of using resilientchild.dk.

**Conclusion:**

The data offer unique opportunities to assess the utilisation of a web-based mental health-promotion intervention following advice from a clinician. This study draws conclusions about which patients are likely to access similar resources and which practice characteristics encourage their use.

**Trial registration:**

Registered in clinicaltrials.gov, Trial number: NCT04129359 Date of registration: 16/10/2019 (https://clinicaltrials.gov/ct2/show/NCT04129359).

## Introduction

Signposting is a way of linking patients in primary care with resources or support within the community, such as web-based interventions or welfare advice [1]. This gives clinicians a non-medical option that can work alongside existing treatments to improve patients’ health and well-being [2]. Signposting is a concept that is still under development, with some countries prioritising signposting as an important part of their general practice forward view [3]. Signposting has become a way of dealing with some of the pressures on general practice arising from demographic change, including increasing life expectancy and birth rates, and rising patient expectations [4, 5]. Despite increasing use of signposting, the evidence base is weak [2, 6] with little consensus around appropriate outcome measures due to the diversity of aims [7].

Social prescribing, which overlaps with signposting but usually involves a formal referral process, can have a limiting effect on the use of urgent and emergency health services, and positively impact people’s well-being [8]. Patients are more likely to enrol if they believe the social prescription will be of benefit, and if the referral is presented in an acceptable way that matches their needs and expectations, with concerns elicited and addressed. Patients are also more likely to engage if their chosen activity is accessible [9]. Signposting to a web-based resource generally involves a looser advisory mechanism, in which clinicians are less likely to receive feedback about their patients’ engagement with the programme. Little is known about whether this approach is beneficial.

The present study takes place in the context of general practitioner (GP)-delivered antenatal care in Denmark. GPs are responsible for three scheduled visits during pregnancy and five postnatal visits, all addressing possible concerns and assessing child development [10]. The content of these scheduled preventive health measures has shifted to some extent from the assessment of physical factors towards psychosocial well-being [10]. In the context of the “Family Well-being In General Practice'' trial, intervention group GPs were asked to signpost a web-based intervention, robustbarn.dk (English: resilientchild.dk) during these scheduled visits, initially at the first antenatal assessment at around 6–10 weeks gestation. Resilientchild.dk is a web-based resource consisting of text, illustrations, and audio material designed to improve parental reflective functioning or mentalisation capacity. This is the capacity to understand and interpret their children’s behaviour and actions in terms of different mental states as well as the parent’s own psychological experiences [11].

## Study aims

To describe participant characteristics and clinic factors associated with the use of resilientchild.dk by pregnant women participating in the trial.

## Methods

### Design

​​This observational study is nested in the “Family Well-being In General Practice'' trial, a cluster randomised trial based in Danish general practice. The trial outcome is child psychological development in the context of scheduled preventive health examinations with an enhanced psychosocial focus and, in the intervention group, signposting towards resilientchild.dk which will be reported in a future paper [12].

### Study setting

The trial (NCT04129359) was initiated in October 2019 and is planned to end in April 2024. Each of the 70 participating practices recruited up to 30 women at their first routine antenatal appointment. In this study, we only analysed data from women in the intervention group because only those were to use resilientchild.dk. Data from 31 practices located in two of five administrative regions in Denmark were analysed. 383 women were recruited for the intervention group. Intervention group GPs were responsible for introducing the women to resilientchild.dk at their first antenatal assessment. After consent, the GP entered the pregnant woman’s social security number into the trial management software REDCap [13]. REDCap then sent an automated invitation to participants’ secure digital post-boxes (eBoks) with a unique login for resilientchild.dk. After the first login, the women created their own password on resilientchild.dk. Women who did not enter the webpage received two written reminders sent to their secure digital post-box.

### Participants

Eligible participants were pregnant women presenting at their first antenatal assessment, usually around 6–10 gestational weeks. Trial exclusion criteria were: lack of Danish literacy, families planning to move to a new general practice during the pregnancy or postnatally, prior participation in the trial with an older child, presentation to the practice later than the third scheduled antenatal visit. Miscarriage or other pregnancy loss after recruitment led to late exclusion [12].

### Intervention (Resilientchild.dk)

Resilientchild.dk is a web-based programme that aims to promote mentalisation skills in parents. The programme was adapted to fit into the context of antenatal care and child health examinations in general practice. Resilientchild.dk can be used by both pregnant women and new families as a problem-solving tool aiming to enhance the parent’s reflective functioning ability. Seventy GP clinics from two of the five Danish regions were recruited by letter and email. In total, 707 pregnant women were informed about the study consecutively from October 2019 until June 2021 by their GP at the first antenatal consultation (between 6 and 10 weeks of gestation). An information sheet was given to the mothers by the GP’s at this consultation including log-in details but formal consent was collected at the time of baseline data collection [14]. Practices in the intervention arm were introduced to resilientchild.dk in a one-day training course in which they were trained to introduce the programme resources at their patient’s first antenatal assessment. In subsequent contacts, GPs in the intervention group were asked to reintroduce resilientchild.dk throughout the scheduled pregnancy and child health examinations when appropriate.

### Intervention usage

Our outcome variable is women’s use of resilientchild.dk in terms of website usage patterns during the first year after entering the study. The women were categorised according to whether they used the intervention or not, as well as how engaged they were in using the intervention. Our final sample was divided into three groups: women categorised as non-users who received the invitation but did not log-in, casual users with fewer than 20 interactions with the website, and engaged users with more than 20 interactions.

## Measures

### Mother characteristics

The following data were collected; mother’s age, occupational status, parity, marital status, Hospital Anxiety and Depression Scale (HADS) [15], Recent Life Event Questionnaire (RLEQ) [16], Adverse Child Experiences Score (ACE) [17] and Prenatal Parental Reflective Functioning Questionnaire (P-PRFQ) [18]. HADS consists of 14 questions, developed to detect states of depression and anxiety [15], and has been extensively used and validated in many countries and settings including general practice [19, 20]. RLEQ aims to assess recent life events occurring in the last 12 months and whether the individual considers the event to be a present influence [16]. ACE is a rating scale that has provided substantial evidence concerning the link between adverse childhood experiences and adult mental and physical illnesses [17]. P-PRFQ is a 14-item questionnaire that aims to assess parental mentalisation in the prenatal period [18].

### General practice characteristics

We collected the following data regarding general practice characteristics; practice organisation categorised as solo GP practice, GP partnership, solo GP sharing facilities, practices with or without a nurse or midwife and how many women each practice had recruited.

### Statistical analysis

The socio-demographic variables and the psychometric scales were compared between the three website use groups by ANOVA (continuously valued variables) or chi-squared tests (categorically valued variables).

**Fig. 1:**
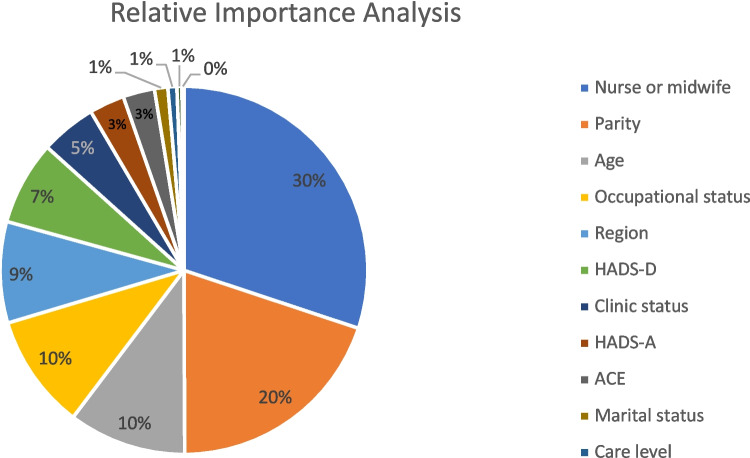
Relative importance analysis

The relative importance of the factors listed in Fig. 1 (Title: Relative importance analysis, legend: Analysis that allows determination of the variables most strongly associated with website use presented as a pie chart) is assessed in a dominance analysis [21]. This analysis divides the coefficient of determination (R^2^) from a full multivariable model into the parts attributable to each of the factors by calculating the mean of the increase in R^2^ adding the corresponding factor to all possible models that may be constructed by the other factors. The statistical model employed in the dominance analysis is an ordinary linear model where the ordinal outcome is treated as a continuous variable. This allows determination of the factors associated with website use.

To assess the direction and magnitude of the influences of the various factors were assessed by adjusted odds-ratios (AOR) from multinomial generalized linear mixed modelling (GLMM). The women are clustered within practices and therefore might be more similar to each other than to those across practices: GLMM accounts for this through a practice random effect. If the women are very similar within practices, ignoring the clustering and claiming they are independent will artificially and erroneously increase the power of the inference; this is particularly true for the practice characteristics that by construction are the same within practice.

In all analyses, non-users were the reference category. Our null hypothesis was that there would be no difference in the characteristics of the women in terms of intervention usage.

The intraclass correlation coefficient (ICC) indicates the proportion of the total variance explained by the grouping structure in the population [22] and is calculated by dividing the random effect variance by the total variance.

All statistical analysis was performed using R (version 4.1.2; R Core Team, 2022).

## Results

230 of the 383 recruited mothers logged onto resilientchild.dk. The active users of the website were matched with the mothers' record-IDs through their email addresses: 198 of the active users were registered in RedCAP, either from the medical pregnancy record or from a previous questionnaire. 42 users of resilientchild.dk were not registered, but 32 of them were matched with an email address by comparing the name of the participating mothers to the names in the addresses of active users. We were unable to match 10 of the email addresses. The final sample consisted of 373 women allocated to the intervention group of the study, which does not include the 10 women for whom we could not match the email addresses. Among our participants, we were able to characterise usage patterns for above 90% and we know that the majority of the participants have accessed the website at least once. With our definition, 143 women were non-users, 113 women were casual users, and 117 women were engaged users.

The socio-demographic characteristics of the women, descriptive statistics, and practice characteristics are summarised in Table 1. The women were between 20 and 49 years of age (mean 31.2). The majority of the women were employed (70.5%), received regular antenatal care (67.6%), were either nulliparous (45.3%) or primiparous (43.7%), and were living with a partner (85.5%). We did not observe any noticeable differences in age distribution between groups.

**Table 1:** Sample characteristics

	Non-user (%)	Casual user (%)	Engaged user (%)	No. of practices (%)	***p***-values
	*n*=143	*n*=113	*n*=117	*n*=31	
**Age mean** (SD)	31.41 (4.6)	31.31 (5.2)	30.94 (4.3)		.71^(ANOVA)^
**Marital status**					.32^(χ2)^
Married	71 (49.6)	50 (44.2)	47 (40.2)		
Not married	61 (42.7)	55 (48.7)	64 (54.7)		
Single	5 (3.5)	8 (7.1)	6 (5.1)		
NA	6 (4.2)	0	0		
**Occupational status**					.19^(χ2)^
Employed	103 (72)	78 (69)	82 (70.1)		
Unemployed	11 (7.7)	7 (6.2)	6 (5.1)		
Sick leave or leave	11 (7.7)	6 (5.3)	5 (4.3)		
Studying	13 (9.1)	22 (19.5)	24 (20.5)		
NA	5 (3.5)	0	0		
**Cohabitation**					.64^(χ2)^
Living with partner	108 (75.5)	102 (90.3)	109 (93.2)		
Living alone	5 (3.5)	8 (7.0)	6 (5.1)		
NA	30 (21)	3 (2.7)	2 (1.7)		
**Parity**					< 0.001*^(χ2)^
Nullipara	51 (35.7)	44 (38.9)	74 (63.2)		
Primipara	71 (49.6)	54 (47.8)	38 (32.5)		
Multipara	21 (14.7)	15 (13.3)	5 (4.3)		
**Care level**					.66^(χ2)^
Regular	98 (68.5)	74 (65.5)	80 (68.4)		
Extended	30 (21.0)	27 (23.9)	32 (27.4)		
NA	15 (10.5)	12 (10.6)	5 (4.2)		
**Region**					.15^(χ2)^
Capital region of Denmark	81 (56.6)	67 (59.3)	70 (59.8)		
Region Zealand	32 (22.4)	43 (38)	45 (38.5)		
NA	30 (21)	3 (2.7)	2 (1.7)		
**HADS**					
HADS-A (SD)	4.29 (3.16)	4.91 (3.63)	4.97 (3.23)		.26^(ANOVA)^
HADS-D (SD)	2.53 (2.24)	3.22 (3.07)	3.62 (2.91)		.014*^(ANOVA)^
**ACE** (SD)	2.76 (2.07)	2.95 (1.83)	2.47 (1.76)		.16^(ANOVA)^
**RLEQ**					
Life events (SD)	2.71 (2.15)	2.60 (2.07)	2.66 (2.30)		.93^(ANOVA)^
Life events that still affect (SD)	0.86 (1.00)	0.81 (1.17)	0.96 (1.55)		.64^(ANOVA)^
**P-PRFQ** (SD)	4.11 (0.88)	3.99 (0.94)	4.08 (0.93)		.58^(ANOVA)^
**Practice type**					
GP partnership				20 (64.5)	
Solo GP				4 (12.9)	
Solo GP sharing facilities				7 (22.6)	
**Nurse or midwife**					
Nurse only				23 (74.2)	
Midwife only				1 (3.2)	
No nurse or midwife				5 (16.1)	
Nurse + midwife				2 (6.5)	

Comparison of characteristics between the women who did and didn’t use the website

Casual user: < 20 interactions on webpage, Engaged user: ≥ 20 interactions on webpage

*SD* Standard deviation, *NA* Not available, *χ2* Chi-sqaure tests, *ANOVA* Analysis of variance

* *p* < 0.05

The sample was generally psychologically robust, scoring low in both HADS, ACE and RLEQ with mean scores of HADS-Anxiety and HADS-Depression being 4.73 (SD 3.35) and 3.14 (SD 2.8) respectively, mean total ACE score 2.73 (SD 1.9) and mean total RLEQ score 2.66 (SD 2.17). The sample demonstrated a moderate level of mentalisation capacity with mean total P-PRFQ score 4.06 (SD 0.9). 50 HADS, 41 RLEQ, 47 P-PRFQ and 44 ACE questionnaires were not completed. In the ANOVA only HADS-D (*p* = 0.016) showed a significant difference between users and non-users. In chi-square tests only parity showed a significant difference between users and non-users (*p* < 0.001).

The relative importance analyses showed that having a nurse or midwife within practice, parity, age, occupational status, and region of residence contributed the most to the likelihood of using the intervention. Results from our relative importance analysis are presented in Fig. 1.

Results from the GLMM are presented in Tables 2 and 3. Table 2 shows the univariate analysis as well as the full multivariate analysis. Our relative importance analysis yielded the five most predictive variables that are included in our final model, shown in Table 3. Our final model showed that parity, whether the clinic had a nurse or midwife, being a student, and region of residence were significant contributors.

**Table 2:** Generalized linear mixed effect model of overall usage

	***Model 1***						***Model 2***					
	*Casual user*			*Engaged user*			*Casual user*			*Engaged user*		
	*p*	*OR*	*95%CI*	*p*	*OR*	*95%CI*	*p*	*OR*	*95%CI*	*p*	*OR*	*95%CI*
***Independent variables***												
**Intercept**												
**Age**												
20-24	0.12	2.69	0.77 – 9.50	0.26	2.18	0.56 – 8.50	0.55	0.58	0.09 – 3.52	0.83	1.21	0.21 – 7.0
25-29	0.57	0.84	0.46 – 1.53	0.60	1.17	0.65 – 2.10	<0.05*	0.33	0.13 – 0.84	0.33	0.63	0.25 – 1.60
30-34	Ref.			Ref.			Ref.			Ref.		
35-39	0.34	1.40	0.70 – 2.83	0.22	1.57	0.77 – 3.18	0.29	1.72	0.64 – 4.59	< 0.05*	3.86	1.20 – 12.36
> 40	0.81	1.15	0.36 – 3.67	0.49	0.60	0.14 – 2.53	0.99	1.01	0.22 – 4.72	0.63	0.66	0.12 – 3.53
**Marital status**												
Married	Ref.			Ref.			Ref.			Ref.		
Not married	0.35	1.28	0.77 – 2.14	0.32	1.90	0.95 – 2.71	0.74	1.14	0.53 – 2.44	0.40	0.72	0.64 – 3.07
Single	0.17	2.27	0.70 – 7.35	0.07	1.60	0.53 – 6.86	0.87	1.14	0.24 – 5.33	0.80	0.77	0.18 – 9.05
**Occupational status**												
Employed	Ref.			Ref.			Ref.			Ref.		
Unemployed	0.73	0.84	0.31 – 2.27	0.45	0.66	0.23 – 1.92	0.30	2.80	0.41 – 19.14	0.54	1.72	0.30 – 10.0
Sick leave or leave	0.54	0.72	0.26 – 2.03	0.32	0.57	0.19 – 1.74	0.73	0.77	0.17 – 3.38	0.84	1.20	0.22 – 6.64
Studying	0.05*	2.23	1.06 – 4.71	< 0.05*	2.34	1.11 – 4.97	< 0.05*	6.30	1.94 – 20.23	< 0.05*	3.74	1.14 – 12.33
**Parity**												
Nullipara	Ref.			Ref.			Ref.			Ref.		
Primipara	0.65	0.88	0.52 – 1.51	< 0.05*	0.33	0.18 – 0.59	0.74	0.87	0.38 – 1.98	< 0.05*	0.26	0.11 – 0.61
Multipara	0.63	0.83	0.38 – 1.80	< 0.05*	0.15	0.05 – 0.44	0.24	0.47	0.13 – 1.65	< 0.05*	0.09	0.02 – 0.43
**Care level**												
Regular	Ref.			Ref.			Ref.			Ref.		
Extended	0.57	1.20	0.65 – 2.17	0.43	1.28	0.69 – 2.38	0.99	1	0.42 – 2.34	0.33	1.60	0.62 – 4.06
**Region**												
Capital region of Denmark	Ref.			Ref.			Ref.			Ref.		
Region Zealand	0.09	1.63	0.93 – 2.85	0.10	1.63	0.92 – 2.89	< 0.05*	2.77	1.18 – 6.48	< 0.05*	4.63	1.68 – 12.69
**Nurse or midwife**												
Nurse	Ref.			Ref.			Ref.			Ref.		
Midwife	0.24	0.26	0.03 – 2.41	0.73	1.26	0.33 – 4.85	1.00	∞		1.00	∞	
Nurse + midwife	0.28	0.60	0.24 – 1.51	0.46	0.72	0.31 – 1.70	0.38	0.58	0.18 – 1.94	0.80	0.84	0.20 – 3.44
No nurse or midwife	0.06	0.53	0.27 – 1.03	< 0.05*	0.32	0.15 – 0.68	< 0.05*	0.22	0.07 – 0.68	<0.05*	0.07	0.01 – 0.34
**Practice type**												
GP partnership	Ref.			Ref.			Ref.			Ref.		
Solo GP sharing facilities	0.08	0.59	0.33 – 1.07	< 0.05*	0.50	0.26 – 0.93	0.66	0.80	0.30 – 2.13	0.42	0.62	0.19 – 1.98
Solo GP practice	0.46	0.69	0.26 – 1.86	0.26	0.54	0.19 – 1.55	0.71	1.40	0.24 – 8.42	0.44	2.39	0.26 – 21.79
**HADS-A**												
0-7	Ref.			Ref.			Ref.			Ref.		
8-10	0.40	1.42	0.63 – 3.19	0.38	1.46	0.62 – 3.44	0.67	1.28	0.40 – 4.20	0.13	2.36	0.77 – 7.23
< 10	0.23	2.14	0.62 – 7.40	0.37	1.84	0.49 – 6.92	< 0.05*	7.85	1.26 – 48.54	0.13	6.36	0.57 – 71.74
**HADS-D**												
0-7	Ref.			Ref.			Ref.			Ref.		
8-10	0.14	5.20	0.60 – 45.32	< 0.05*	9.87	1.20 – 81.04	0.40	2.80	0.25 – 31.58	0.21	5.31	0.38 – 74.07
> 10	0.40	2.08	0.37 – 11.62	0.61	1.63	0.25 – 10.67	0.61	0.49	0.03 – 8.07	0.45	0.33	0.02 – 5.76
**ACE**												
< 4	Ref.			Ref.			Ref.			Ref.		
**>** 4	0.28	1.38	0.77 – 2.44	0.44	0.77	0.80 – 1.07	0.64	1.21	0.54 – 2.75	0.22	0.57	0.23 – 1.40
**RLEQ**												
Life events	0.69	0.97	0.86 – 1.11	0.91	0.99	0.88 – 1.12	0.85	1.02	0.83 – 1.26	0.88	0.98	0.80 – 1.21
Events that still affects the individual	0.75	0.96	0.75 – 1.22	0.51	1.07	0.87 – 1.32	0.30	0.81	0.54 – 1.20	0.94	0.99	0.68 – 1.44
**P-PRFQ**	0.32	0.86	0.64 – 1.16	0.87	0.97	0.71 – 1.33	0.52	0.88	0.58 – 1.31	0.81	0.95	0.61 – 1.47

Results from the univariate analysis is presented in model 1, and results from the multivariate analysis is presented in model 2

The table shows p-values, odds ratios (OR) and 95% confidence intervals (CI) for both casual users and engaged users

Casual user: < 20 interactions on webpage, Engaged user: ≥ 20 interactions on webpage

*HADS-A* Hospital Anxiety and Depression Scale-Anxiety, *HADS-D* Hospital Anxiety and Depression Scale-Depression, *ACE* Adverse Childhood Experiences Questionnaire, *RLEQ* Recent Life Events Questionnaire, *P-PRFQ* Prenatal-Parental Reflective Functioning Questionnaire

*: *p* < 0.05

**Table 3:** Generalized linear mixed effect model of overall usage

	***Model 3***					
	*Casual user*			*Engaged user*		
***Independent variables***	*p*	*OR*	*95% CI*	*p*	*OR*	*95% CI*
**Age**						
20-24	0.89	1.11	0.24 – 5.12	< .05*	0.95	0.19 – 4.62
25-29	0.05	0.48	0.23 – 0.99	0.10	0.54	0.26 – 1.12
30-34	Ref.	Ref.	Ref.	Ref.	Ref.	Ref.
35-39	0.26	1.60	0.71 – 3.58	< .05*	2.48	1.03 – 5.92
> 40	0.92	0.94	0.28 – 3.14	0.57	0.59	0.13 – 2.71
**Parity**						
Nullipara	Ref.			Ref.		
Primipara	0.25	0.68	0.36 – 1.30	< .05*	0.23	0.12 – 0.46
Multipara	0.35	0.64	0.24 – 1.65	< .05*	0.08	0.02 – 0.29
**Nurse or midwife**						
Nurse	Ref.	Ref.	Ref.	Ref.	Ref.	Ref.
Midwife	0.26	0.25	0.02 – 2.84	0.73	1.32	0.27 – 6.48
Nurse + midwife	0.34	0.60	0.21 – 1.70	0.50	0.69	0.23 – 2.01
No nurse or midwife	< .05*	0.43	0.20 – 0.93	< .05*	0.17	0.07 – 0.44
**Occupational status**						
Employed	Ref.			Ref.		
Unemployed	0.50	1.53	0.44 – 5.28	0.57	0.69	0.20 – 2.45
Sick leave or leave	0.74	0.83	0.27 – 2.56	0.84	0.87	0.24 – 3.23
Studying	< .05*	3.40	1.34 – 8.57	0.12	2.08	0.83 – 5.26
**Region**						
Capital region of Denmark	Ref.	Ref.	Ref.	Ref.	Ref.	Ref.
Region Zealand	0.05	1.87	0.99 – 3.51	< .05*	2.40	1.22 – 4.76

Results from the analysis with the five most contributive variables derived from the relative importance analysis is presented in model 3

The table shows p-values, odds ratios (OR) and 95% confidence intervals (CI) for both casual users and engaged users

Casual user: < 20 interactions on webpage, Engaged user: ≥ 20 interactions on webpage

* *p* < 0.05

Parous women were less likely to be engaged users compared to non-users, both for primiparous and multiparous women compared to nulliparous. The AOR favoured a negative relationship with a 77% and 92% decrease respectively. It was also found that if the practice did not employ a nurse or midwife the women were more likely to be non-users. The AOR favoured a negative relationship with a 57% and an 83% decrease respectively. The model also shows that the women are more likely to be casual users compared to non-users if they were studying compared to those who are working. The AOR suggests that students were 3.4 times more likely to be casual users. Finally, the model shows that the women are more likely to be engaged users compared to non-user if they were living in region Zealand compared to living in the Capital region of Denmark: women living in region Zealand were 2.4 times more likely to be engaged users.

ICC was low for all of our models, which is often seen in clustering in primary care [23], suggesting that the characteristics of women clustered in practices were not highly similar.

## Discussion

These results demonstrate that there are two main drivers of the use of a preventive web-based intervention; 1) if the GP had a midwife or nurse involved in the project. 2) Parity of the women.

We observed that women attending clinics that did not have a midwife or nurse dedicated to antenatal care were less active users of the intervention webpages. This might relate to the professional identity of midwifes and nurses, seeing the nature of the intervention (promoting a healthy mother-infant relationship) as linked to the core values of their profession [24, 25].

The website requires time to fully engage in the intervention, and this may be easier if participants have no existing childcare responsibilities and are supported by a partner. In line with this hypothesis, we found that nulliparous women living with a partner are more likely to use the intervention.

This has been a unique opportunity to assess the utilisation of web-based interventions signposted by GPs and to investigate characteristics associated with taking up the signposting message. The resilientchild.dk intervention may be a useful resource that could routinely be introduced at antenatal assessments, but the results we present may well be relevant to signposting towards any health promotion intervention.

Studies regarding web-based interventions have given us valuable information: web-based interventions that target specific mental conditions have shown good results [26] with studies showing significantly lower levels of depressive symptoms [27] as well as increased rates of remission from already diagnosed depression. We also know that web-based interventions can lead to a significantly more rapid reduction in the severity of depressive symptoms [28] and significant overall reductions in anxiety and psychological distress [29]. Nevertheless, it has been proposed that targeting high-risk groups is not sufficient and that a population-based approach that includes low-risk groups is essential to optimising long term benefit [30]. Positive psychological interventions that try to enhance subjective mental well-being (SWB) are often considered a parallel to mental health promotion and treatment. Positive psychological interventions have been shown to improve SWB and help reduce depressive symptoms [31], which can make them a relevant tool in a population-based approach.

Signposting has the potential to release some of the pressure on general practice but there is still a need for more studies to improve the evidence foundation underlying signposting and its effectiveness. There are numerous ways that physicians can advise patients towards non-medical resources, and signposting should not be thought of as a single intervention but as an approach that can be used in different settings. This can also create challenges, as the generalisable claims can be limited due to many different outcomes and difficulties defining effectiveness. This does however strengthen the case for more studies that use this approach to expand the evidence foundation [9, 32].

### Strengths and limitations

To our knowledge, this is one of the first reports of the uptake of signposting to web-based general health promotion in general practice. The models from our statistical analysis give us an opportunity to generalise many of our findings to a larger population. This study tries to build upon the evidence base underlying signposting and provides information about participant characteristics that could be useful when developing new resources related to signposting or evaluating other ongoing resources related to signposting.

Several limitations of our study need to be acknowledged. First, our sample was relatively privileged, with the majority of the women being married, employed, and living with a partner, which could indicate a higher level of opportunity to engage in research studies like this. Furthermore, most of the women that were included lived in relatively affluent areas in the urban capital region of Denmark. All of these are factors that could limit the generalisability of the study. Our sample is also exposed to selection bias. The practices that participated have an interest in pregnancy and paediatrics. Within-practice bias is also apparent since not everybody will be invited to the study, and some that were invited did not accept [33]. It is also likely that there would be selective attrition of families with problems as the study progressed [34].

Second, we did not have information about educational background available, which might have been useful in interpreting the results. It is possible that women with higher educational levels might be more likely to use an intervention like this. Information about income was also not available. Occupational status was therefore our only measure of socioeconomic status.

Third, we used several questionnaires in our study. Each questionnaire is time-consuming and could potentially feel unmanageable, possibly more likely among women with low levels of resources.

Fourth, our sample size at practice level in our GLMM analysis was small at 31. Sample sizes of 50 or less may lead to biased estimates of the standard errors. Estimates of regression coefficients are however unbiased [35].

Finally, we were unable to match ten emails that were not registered in RedCAP, so it is possible that up to ten individuals classified as non-users should have been included in the user group.

## Conclusion

This study has elucidated factors that are most likely to be associated with the use of an online resource, signposted in general practice. This knowledge may be useful when developing and evaluating interventions that involve signposting to web-based resources. Web-based signposting, appropriately tailored, could be a valuable tool for clinicians wishing to promote psycho-social well-being but there are still major gaps in our knowledge about when, how, and for whom these interventions are likely to be effective.

## Data Availability

The dataset generated in this study cannot be publicly shared due to privacy issues as the dataset contain protected health information concerning the participants. A redacted dataset can however be made available by the corresponding author upon reasonable request. Informed consent to participate was obtained from all participants.

## References

[1] Lisa B, Abigail T, Jane F, Damian H, Pauline AN. The challenges of integrating signposting into general practice: qualitative stakeholder perspectives on care navigation and social prescribing in primary care. BMC Primary Care. 2022. 10.1186/s12875-022-01669-z PMC8972897 35365072

[2] Bickerdike L, Booth A, Wilson PM, Farley K, Wright K (2017). Social prescribing: less rhetoric and more reality. A systematic review of the evidence. BMJ Open.

[3] England N. General Practice Forward View 2016 [Available from: https://www.england.nhs.uk/wp-content/uploads/2016/04/gpfv.pdf.

[4] Brandling J, House W (2009). Social prescribing in general practice: adding meaning to medicine. Br J Gen Pract.

[5] Pescheny JV, Randhawa G, Pappas Y (2019). The impact of social prescribing services on service users: a systematic review of the evidence. Eur J Pub Health.

[6] England PH. Effictiveness of social prescribing: An evidence synthesis PHE Publications2019 [Available from: https://ukhsa.koha-ptfs.co.uk/cgi-bin/koha/opac-retrieve-file.pl?id=9c033e58d33d6eb6f59dae978c0f7839.

[7] Rempel E, Wilson E, Durrant H, Barnett J (2017). Preparing the prescription: A review of the aim and measurement of social referral programmes. BMJ Open.

[8] University SH. The Rotherham Social Prescribing Service for People with Long-Term Health Conditions 2015 [Available from: https://shura.shu.ac.uk/17296/1/rotherham-social-prescribing-service-annual-report.pdf.

[9] Husk K, Blockley K, Lovell R, Bethel A, Lang I, Byng R (2020). What approaches to social prescribing work, for whom, and in what circumstances? A realist review. Health Soc Care Community.

[10] Sundhedsstyrelsen. Anbefalinger for svangreomsorgen 2021 [Available from: https://www.sst.dk/-/media/Udgivelser/2021/Anbefalinger-svangreomsorgen/Anbefalinger-for-svangreomsorgen.ashx.

[11] Slade A (2005). Parental reflective functioning: an introduction. Attach Hum Dev.

[12] ClinicalTrials.gov USNLoM. FamilieTrivsel i Almen Praksis: a Mentalisation Programme for Families With Young Children [Available from: https://clinicaltrials.gov/ct2/show/NCT04129359.

[13] Harris PA, Taylor R, Thielke R, Payne J, Gonzalez N, Conde JG (2009). Research electronic data capture (REDCap)–a metadata-driven methodology and workflow process for providing translational research informatics support. J Biomed Inform.

[14] Overbeck G, Kragstrup J, Gørtz M, Rasmussen IS, Graungaard AH, Siersma V (2023). Family wellbeing in general practice: a study protocol for a cluster-randomised trial of the web-based resilience programme on early child development. Trials.

[15] Zigmond AS, Snaith RP (1983). The hospital anxiety and depression scale. Acta Psychiatr Scand.

[16] Health UKDo. Recent Life Events Questionnaire [Available from: https://proceduresonline.com/trixcms/media/4898/recent-life-event-questionnaire.pdf.

[17] Felitti VJ, Anda RF, Nordenberg D, Williamson DF, Spitz AM, Edwards V (1998). Relationship of childhood abuse and household dysfunction to many of the leading causes of death in adults. The Adverse Childhood Experiences (ACE) Study. Am J Prev Med.

[18] Pajulo M, Tolvanen M, Karlsson L, Halme-Chowdhury E, Öst C, Luyten P (2015). The prenatal parental reflective functioning questionnaire: Exploring factor structure and construct validity of a new measure in the finn brain birth cohort pilot study. Infant Ment Health J.

[19] Bjelland I, Dahl AA, Haug TT, Neckelmann D (2002). The validity of the Hospital Anxiety and Depression Scale. An updated literature review. J Psychosom Res.

[20] Herrmann C (1997). International experiences with the Hospital Anxiety and Depression Scale–a review of validation data and clinical results. J Psychosom Res.

[21] Johnson JW, Lebreton JM (2004). History and Use of Relative Importance Indices in Organizational Research. Organ Res Methods.

[22] Leyland AH, Groenewegen PP. Multilevel Modelling for Public Health and Health Services Research Health in Context. 1st 2020. ed. Cham: Springer International Publishing; 2020. 33347097

[23] Singh J, Liddy C, Hogg W, Taljaard M (2015). Intracluster correlation coefficients for sample size calculations related to cardiovascular disease prevention and management in primary care practices. BMC Res Notes.

[24] Brewington J, Godfrey N (2020). The Professional Identity in Nursing Initiative. Nurs Educ Perspect.

[25] Caza BB, Creary S (2016). Perspectives on Contemporary Professional Work.

[26] Andrews G, Basu A, Cuijpers P, Craske MG, McEvoy P, English CL (2018). Computer therapy for the anxiety and depression disorders is effective, acceptable and practical health care: An updated meta-analysis. J Anxiety Disord.

[27] Forsell E, Bendix M, Hollandare F, Szymanska von Schultz B, Nasiell J, Blomdahl-Wetterholm M (2017). Internet delivered cognitive behavior therapy for antenatal depression: A randomised controlled trial. J Affect Disord.

[28] Milgrom J, Danaher BG, Gemmill AW, Holt C, Holt CJ, Seeley JR (2016). Internet Cognitive Behavioral Therapy for Women With Postnatal Depression: A Randomized Controlled Trial of MumMoodBooster. J Med Internet Res.

[29] Loughnan SA, Sie A, Hobbs MJ, Joubert AE, Smith J, Haskelberg H (2019). A randomized controlled trial of 'MUMentum Pregnancy': Internet-delivered cognitive behavioral therapy program for antenatal anxiety and depression. J Affect Disord.

[30] Huppert FA (2009). A New Approach to Reducing Disorder and Improving Well-Being. Perspect Psychol Sci.

[31] Bolier L, Haverman M, Westerhof GJ, Riper H, Smit F, Bohlmeijer E (2013). Positive psychology interventions: a meta-analysis of randomized controlled studies. BMC Public Health.

[32] Husk K, Elston J, Gradinger F, Callaghan L, Asthana S (2019). Social prescribing: where is the evidence?. Br J Gen Pract.

[33] Ertmann RK, Nicolaisdottir DR, Kragstrup J, Siersma V, Overbeck G, Wilson P (2020). Selection bias in general practice research: analysis in a cohort of pregnant Danish women. Scand J Prim Health Care.

[34] Wolke D, Waylen A, Samara M, Steer C, Goodman R, Ford T (2009). Selective drop-out in longitudinal studies and non-biased prediction of behaviour disorders. Br J Psychiatry.

[35] Maas CJM, Hox JJ (2005). Sufficient Sample Sizes for Multilevel Modeling. Methodology.

